# Development of a Twitter-Based Intervention for Smoking Cessation that Encourages High-Quality Social Media Interactions via Automessages

**DOI:** 10.2196/jmir.3772

**Published:** 2015-02-23

**Authors:** Cornelia Pechmann, Li Pan, Kevin Delucchi, Cynthia M Lakon, Judith J Prochaska

**Affiliations:** ^1^University of California IrvineThe Paul Merage School of BusinessIrvine, CAUnited States; ^2^Shanghai Jiao Tong UniversityAntai College of Economics and ManagementShanghaiChina; ^3^University of California San FranciscoDepartment of PsychiatrySan Francisco, CAUnited States; ^4^University of California IrvineProgram in Public HealthIrvine, CAUnited States; ^5^Stanford UniversityStanford Prevention Research CenterStanford, CAUnited States

**Keywords:** smoking cessation, social media, text messaging

## Abstract

**Background:**

The medical field seeks to use social media to deliver health interventions, for example, to provide low-cost, self-directed, online self-help groups. However, engagement in online groups is often low and the informational content may be poor.

**Objective:**

The specific study aims were to explore if sending automessages to online self-help groups encouraged engagement and to see if overall or specific types of engagement related to abstinence.

**Methods:**

We conducted a Stage I Early Therapy Development Trial of a novel social media intervention for smoking cessation called Tweet2Quit that was delivered online over closed, 20-person quit-smoking groups on Twitter in 100 days. Social media such as Twitter traditionally involves non-directed peer-to-peer exchanges, but our hybrid social media intervention sought to increase and direct such exchanges by sending out two types of autocommunications daily: (1) an “automessage” that encouraged group discussion on an evidence-based cessation-related or community-building topic, and (2) individualized “autofeedback” to each participant on their past 24-hour tweeting. The intervention was purposefully designed without an expert group facilitator and with full automation to ensure low cost, easy implementation, and broad scalability. This purely Web-based trial examined two online quit-smoking groups with 20 members each. Participants were adult smokers who were interested in quitting and were recruited using Google AdWords. Participants’ tweets were counted and content coded, distinguishing between responses to the intervention’s automessages and spontaneous tweets. In addition, smoking abstinence was assessed at 7 days, 30 days, and 60 days post quit date. Statistical models assessed how tweeting related to abstinence.

**Results:**

Combining the two groups, 78% (31/40) of the members sent at least one tweet; and on average, each member sent 72 tweets during the 100-day period. The automessage-suggested discussion topics and participants’ responses to those daily automessages were related in terms of their content (*r*=.75, *P*=.012). Responses to automessages contributed 22.78% (653/2867) of the total tweets; 77.22% (2214/2867) were spontaneous. Overall tweeting related only marginally to abstinence (OR 1.03, *P*=.086). However, specific tweet content related to abstinence including tweets about setting of a quit date or use of nicotine patches (OR 1.52, *P*=.024), countering of roadblocks to quitting (OR 1.76, *P*=.008) and expressions of confidence about quitting (OR 1.71, SE 0.42, *P*=.032). Questionable, that is, non-evidence-based, information about quitting did not relate to abstinence (OR 1.12, *P*=.278).

**Conclusions:**

A hybrid social media intervention that combines traditional online social support with daily automessages appears to hold promise for smoking cessation. This hybrid approach capitalizes on social media’s spontaneous real-time peer-to-peer exchanges but supplements this with daily automessages that group members respond to, bolstering and sustaining the social network and directing the information content. Highly engaging, this approach should be studied further.

**Trial Registration:**

Clinicaltrials.gov NCT01602536; https://clinicaltrials.gov/ct2/show/NCT01602536 (Archived by WebCite at http://www.webcitation.org/6WGbt0o1K)

## Introduction

The medical field is increasingly interested in using social media such as Facebook and Twitter for delivering health interventions, including interventions that involve peer-to-peer social support and information exchange such as online self-help groups [1]. Social media holds promise for delivering health interventions because it is popular; 73% of online adults reportedly use social media with 42% using multiple sites and often daily [2]. Also, social media is virtually free to users, interactive, and accessible 24-7 to anyone with a mobile phone, tablet, or computer connected to the Internet.

Twitter seems especially promising for facilitating online self-help groups because it allows users to send short messages or “tweets” of up to 140 characters instantly to multiple others and to receive immediate feedback from one or many. Moreover, private Twitter groups can be set up in which all members, and only members, can simultaneously see and reply to the posts. Group members can post photographs further encouraging intimate relationships and online communities to form [3,4].

In addition, Twitter’s user-friendly application programming interface (API) facilitates health care and research applications. Software programs written for Twitter can send out “autocommunications” or pre-scheduled automatically delivered communications as Twitter posts, or alternatively as mobile texts or emails. Other programs written for Twitter can download past tweets into searchable databases, and all of this has substantial utility for supporting and understanding health behavior change. As a result, there is an abundance of health and medical applications of Twitter [1].

Nevertheless, there are concerns about using social media-based health interventions on Twitter or elsewhere. An overriding concern is that engagement or interactivity may be low [5-8]. For example, though millions of people use health forums to obtain health information, most users do not post frequently or continually and so effects on health outcomes have been modest [6]. However, people who actively engage online often do benefit [9,10]. Thus the underlying problem seems to be low engagement [11], which is potentially correctable by finding mechanisms to increase engagement [4,11]. For instance, sending daily automessages to online self-help groups that suggest group discussion topics might possibly trigger engagement.

Another concern about using social media for health interventions and especially for online self-help groups is that peer-to-peer postings may be of poor quality, for example, inconsistent with clinical practice guidelines [12,13]. In fact, many studies have documented questionable postings [14,15]. For instance, a study of Twitter accounts identified using the search criteria of “quit or stop smoking” found a preponderance of use by bloggers to promote non-evidence-based cessation products such as e-cigarettes, herbs, and lasers [14]. Sending daily automessages to online self-help groups might also possibly help with the problem of non-evidence-based content, by directing the information content toward evidence-based topics.

The current research builds on past studies of one-way automessage-based health interventions, that is, interventions involving text or email messages created by health experts that are automatically sent out to individual recipients via computers on fixed schedules to encourage health behaviors. Several initial studies indicated that automessage interventions were effective for smoking cessation [16-20], but a recent review found that just three of 15 randomized trials of automessage-based smoking cessation interventions demonstrated a significant improvement over control [21]. Sending daily automessages to online quit-smoking groups that encourage evidence-based information exchange and social support might possibly increase intervention efficacy, and so our study takes a first look at this.

The current research involved a Stage I Early Therapy Developmental Trial [22] of a novel social media-based intervention for smoking cessation called Tweet2Quit. This was a hybrid social media intervention because traditional social media like Twitter is characterized by non-directed peer-to-peer exchanges, and we sought to encourage and direct such exchanges with our daily autocommunications. Using automated software programs that ran off our study website, we sent out daily automessages to bolster and sustain the peer-to-peer exchanges and to encourage evidence-based discussion topics, and we sent out daily individualized autofeedback on prior-day tweeting.

Consistent with the call for N-of-1 or small-scale trials that target discrete but significant questions for advancing mHealth interventions [23,24], this initial trial tested the Tweet2Quit intervention in two pilot groups of 20 members each. To assess the possible social media-based behavior change mechanisms, we coded participants’ tweets on two dimensions: (1) whether or not the tweet was an automessage-generated response, that is, a response to an intervention automessage, and (2) the specific content of the tweet. To examine the potential merits of our hybrid approach of combining peer support with automessages, we formulated two specific research aims: (1) to explore whether the automessages encouraged engagement, that is, tweeting, and (2) to assess if overall engagement or specific types of engagement were related to abstinence. Due to the nonrandomized treatment-only research design, statistical models were used that included three participant-level covariates that have been found to relate to abstinence success in the literature: education [25-28], gender [26,29], and baseline cigarettes per day [27,30,31].

## Methods

### Overview

We conducted a developmental trial of the Tweet2Quit intervention by setting up two consecutive online quit-smoking groups with 20 adult smokers per group (total N=40). Recruitment, screening, informed consent, assessments (baseline, 7, 30, and 60 days), and intervention delivery all occurred online. The research took place in 2012 with approval and oversight from the Institutional Review Boards of the three sponsoring US universities. As a main function of the developmental trial, in Group 1 we identified improvements in participant screening, intervention delivery, and assessment methods that we implemented in Group 2.

### Twitter-Based Intervention

Tweet2Quit was delivered using closed, 20-person, 100-day peer-to-peer Twitter support groups. The decision to include 20 smokers in each group was based on research indicating that a typical virtual social network has about 17-20 active participants [32-34]. The intervention combined (1) a daily “automessage” that appeared as a Twitter post and posed a question to encourage a group-level discussion on an evidence-based cessation-related topic [12,13] or community-building topic [3,4], and (2) daily individualized “autofeedback” that either praised engagement or encouraged more engagement based on past 24-hour tweeting.

The intervention was purposefully developed without an expert group moderator in order to be low cost, fully automated, completely scalable, real-time, and peer-to-peer. We selected Twitter as the social media platform over Facebook because it was easier to keep posts private, that is, within the group, relative to Facebook and also because the Twitter programming language is superior. We set up the groups to be private to ensure confidentiality. That is, we set up each group member to follow and be followed exclusively by the other members, and we instructed members not to let others join.

### Twitter Set-Up

We created new email and Twitter accounts for the study participants because this allowed us to access the accounts if a participant’s tweeting behavior became problematic, although this never occurred. Participants provided their own preferred usernames and passwords. We sent participants simple instructions to set up their mobile phones to send and receive texts from Twitter because this required physical access to their phones. We encouraged participants to post a photo or image for personalization of their Twitter account. Most importantly, we encouraged participants to tweet their group daily, reiterating this multiple times during recruitment, screening, and group assignment, and in the daily autocommunications. Participants often directed their tweets to one or more specific group members using the @ sign, and over time numerous social dyads and triplets formed (CM Lakon et al, unpublished data, 2015), but Twitter automatically sent every tweet simultaneously to all group members ensuring their full access to all posts at all times.

### Intervention Autocommunications

A novel part of the intervention was the development of daily automessages suggesting discussion topics that were posed as questions to prompt tweeting. The automessages were sent out mechanically from the study website using a Twitter-based software program. The messages came from an account labeled “smokingcessat” and were posted as tweets, that is, they appeared on the group’s Twitter feed. Most of the automessages encouraged discussions that were consistent with clinical practice guidelines for smoking cessation [12,13] and referred to the functional, emotional, and/or self-identity benefits of quitting smoking [35,36]. Additional automessages promoted group bonding, that is, online community formation [3,4]. As a main function of our developmental trial, based on our initial learnings, we increased the number and improved the timing of the automessages from Group 1 to Group 2.

Table 1 summarizes the complete set of 100 automessages that were used in Group 2. A similar or representative subset of these automessages, totaling 58, was used in Group 1. The automessages encouraged participants to share their smoking histories or other personal information (23%), identify rewards for quitting (19%), counter roadblocks to quitting (13%), identify roadblocks to quitting (9%), express emotional support for quitting (9%), set a quit date or use nicotine patches (6%), or express confidence about quitting (5%). Some of the automessages asked about the intervention (16%) at the intervention end.

**Table 1:** Automessage topics versus automessage-generated tweets^a^.

Automessage topics	Verbatim examples	Main benefit to participants	Percent of automessages (N=100), % (n)	Percent of automessage-generated tweets (N=653), % (n)
Sharing of smoking histories or other personal information	How many years did you smoke?	Self-identity	23.0 (23)	38.0 (248)
Identification of rewards for quitting	How do you reward yourself for being a nonsmoker each day?	Emotional	19.0 (19)	18.1 (118)
Countering of roadblocks to quitting	What will you do when you feel the urge to smoke?	Functional	13.0 (13)	3.1 (20)
Identification of roadblocks to quitting	What activities, responsibilities, tasks, or people were or are the biggest triggers for you to smoke?	Functional	9.0 (9)	8.0 (52)
Expressions of emotional support for quitting	Many of you have quit smoking for an entire month! Congratulations! How does it feel?	Emotional	9.0 (9)	3.0 (19)
Setting of a quit date or use of nicotine patches	How do you remind yourself to put on a new patch each day?	Functional	6.0 (6)	2.0 (13)
Expressions of confidence about quitting	Do you feel confident that you are now a nonsmoker?	Self-identity	5.0 (5)	6.0 (39)

^a^16.0% (16/100) of automessages asked about the intervention, eliciting 11.0% (72/653) of automessage-generated tweets. No automessages asked for questionable information about quitting or assertions of abstinence but, of the tweets coded as automessage-generated due to their timing, 6.0% (39/653) and 5.1% (33/653) were coded as containing such content, respectively.

Group 1 received daily automessages for the first 30 days followed by automessages 3x/week for 70 days, and these automessages were sent out at nighttime (12 a.m. Pacific, 3 a.m. Eastern) to stimulate a response the next morning. Analyses, however, indicated that the nighttime automessage timing was suboptimal because there were no spikes in tweeting the next morning (details below), and so the timing was changed. Furthermore, when Group 1’s automessages were reduced to 3x/week, their tweeting declined markedly. Thus Group 2 received one automessage per day for the full 100 days, and these automessages were sent out in the evening (5 p.m. Pacific, 8 p.m. Eastern) to stimulate an immediate response.

### Intervention Autofeedback Sent via Twitter

To further encourage engagement, each morning for 100 days (9 a.m. Pacific, 12 p.m. Eastern), participants received daily autofeedback on their prior 24-hour tweeting behavior from the study website. A Twitter-based software program automatically downloaded the tweets every night, identified tweeters and non-tweeters, and sent prewritten autofeedback praising tweeters for engaging and encouraging non-tweeters to engage using varied wording. In Group 1, the autofeedback was posted on the group’s Twitter feed, but we learned that many participants were not logging onto Twitter and so they were not receiving the autofeedback. Hence in Group 2, the autofeedback was sent out as texts to each participant’s mobile phone to reach those not logged onto Twitter. We had initially planned to cease all autocommunications at 60 days when the free nicotine patches and abstinence surveys ended. However, many of the Group 1 participants kept tweeting past 60 days, and so we continued the autocommunications through day 100 in both groups.

### Nicotine Patches and Quit Date

Each participant was mailed an 8-week supply of nicotine patches that was dosed per the baseline smoking level (starting with 14 mg patches if <10 cigarettes/day and 21 mg patches if >10 cigarettes/day). Participants were instructed to initiate patch use on their quit date. Clinical practice guidelines recommend combined pharmacological and behavioral treatment to address the physiological and psychological components of nicotine addiction in regular daily smokers [13].

In addition, participants were referred to the National Institutes of Health online quit-smoking guide to develop a quit plan and were instructed to set a quit date and initiate patch use on their quit date. Group 1 participants were instructed to set a quit date that was within 14 days of intervention start based on clinical practice guidelines [13]. We found, however, that those who delayed setting a quit date until the second week also delayed engaging with the group and were marginalized by the group. Thus for Group 2, the quit window was reduced to within 7 days of intervention start.

### Sample Recruitment and Screening

Smokers were recruited using Google AdWords and a US $2,000/month pro bono ad budget provided by the Bonnie J. Addario Lung Cancer Foundation. When a person typed a cessation keyword into a Google search (eg, nicotine patches, quit smoking), a study ad appeared if our automated ad bid (maximum of $2/keyword) exceeded competing bids. The Google ads linked to the Tweet2Quit website that provided study information and a brief application form. Recruitment took about 4 months per group.

Applicants were contacted by email about 1 month prior to study start and given a link to the screening survey that also included the informed consent form. Exclusion criteria included contraindications to nicotine patch use; active prescription medicine for depression, anxiety, or quitting smoking; use of an illicit hard drug in the past 4 weeks; or residency with another participant. The inclusion criteria were smoked 100+ cigarettes in one’s lifetime, currently smoking 5+ cigarettes per day, intending to quit in the next month, aged 18-59, English speaking, and continental US resident with an active email account, mobile phone with Internet access and unlimited texting, and weekly texting. For Group 2, daily Facebook use was added as an inclusion criterion because this related significantly to Group 1 participants’ tweeting volume. Also for Group 2, daily marijuana use was added as an exclusion criterion because a Group 1 member reported using marijuana daily to avoid tobacco and recommended this to others.

### Survey Measures

The baseline survey assessed participants’ age, gender, ethnicity, marital status, education, and smoking history, and it included the Fagerstrom Test of Nicotine Dependence [37]. The primary outcome, smoking abstinence, was assessed at 7, 30, and 60 days after the quit date that participants had recorded on the study website. In both groups, 25% (10/40) of participants chose day 1 as their quit date while 75% (30/40) chose a later date. Three participants, all from Group 1, failed to enter a quit date and were given the last possible date.

At each assessment point, abstinence was measured using two standard self-report questions about 7-day point prevalence smoking: “How many cigarettes have you smoked in the past 7 days?” and “Have you puffed on a cigarette within the past 7 days?” Any smoking or puffing was recorded as non-abstinent. Non-responses were recorded as missing. As a secondary outcome and an indicator of treatment adherence, we also measured participants’ nicotine patch use (yes/no). We measured this for the past week at the 7-day follow-up, and for the past month at the 30-day and 60-day follow-ups. Non-responses were recorded as missing.

In Group 1, the follow-up assessments were conducted via emailed links to online surveys, but response rates were lower than expected: 60%, 65%, and 60% at 7, 30, and 60 days post quit date, respectively. So for Group 2, we also sent texts and called by phone to obtain the survey responses, and response rates improved to 95%, 90% and 80%, respectively.

### Tweeting Measures

The groups’ tweets for each day were automatically downloaded to an Excel database using another Twitter software program, and we assessed tweeting volume, content, and timing as secondary outcomes. The database contained a separate record for each tweet that showed the verbatim message sent, the sender’s username, each recipient’s username if designated (eg, by @), and the date and time. We then summed the tweets by group, participant, week of study, and time of day. We also recorded whether each participant tweeted at least once and continued tweeting past day 30.

Furthermore, the tweets were content coded based on the discussion topics that were posed in the automessages and based on other common discussion topics as reflected in the tweets. A codebook was created with 15 mutually exclusive and collectively exhaustive content codes, and each tweet received a single code (see Table 2). Furthermore, the tweets were coded to indicate whether they were in response to an automessage or spontaneous. Automessage-generated responses or tweets were identified based on whether the tweet was addressed to the account “smokingcessat” that sent the automessage and/or occurred shortly after the automessage was sent and was associated with the question posed. All other tweets were coded as spontaneous.

Two trained research assistants independently coded the tweets. For the tweet content coding, the kappa or intercoder reliability was .94 for Group 1 (95% CI 0.93-0.96) and .80 for Group 2 (95% CI 0.78-0.82). For the coding of automessage-generated versus spontaneous tweets, the kappa was .86 for Group 1 (95% CI 0.79-0.94) and .91 for Group 2 (95% CI 0.88-0.93).

**Table 2:** Total tweets and spontaneous tweets by topic and abstinence^a^.

Overall tweet topics	Verbatim examples	Main benefit	Total tweets (N=2867), % (n)	Spontaneous tweets (N=2214, 77%), % (n)	Relationship between total tweets and abstinence	
					OR (SE)	*P* value
Sharing of smoking histories or other personal information	I'm a mom of 4, just got married a month ago	Self-identity	24.00 (688)	20.01 (443)	1.08 (0.07)	.237
Expressions of emotional support for quitting	Day 2 for you? Hang in there...it gets easier!!	Emotional	22.01 (631)	28.00 (620)	1.04 (0.03)	.156
Assertions of abstinence	@jenjencan I have been 32 hours without it after the last 18years!!!!	Self-identity	12.00 (344)	14.00 (310)	1.17 (0.09)	.031
Identification of roadblocks to quitting	Anyone else smoke when they drive alone? I have a 30-55 min commute each way to work, usually smoke 2x b4 arrival. Ideas to fight the urge?	Functional	10.01 (287)	10.00 (221)	1.02 (0.08)	.754
Identification of rewards for quitting	My goal after quitting in playing in local tennis tournament and hope i make it past first round.	Emotional	8.00 (229)	5.01 (111)	1.26 (0.16)	.065
Sharing of questionable information about quitting	each time i want to grab for a smoke i eat a single piece of candy	Emotional	6.00 (172)	6.01 (133)	1.12 (0.11)	.278
Setting of a quit date or use of nicotine patches	Set my date for 1/21	Functional	4.01 (115)	5.01 (111)	1.52 (0.28)	.024
Countering of roadblocks to quitting	I'm doing yoga and chewing straws to cope, what is everyone else doing?	Functional	3.00 (86)	2.98 (66)	1.76 (0.37)	.008
Expressions of confidence about quitting	I quit once before so I'm counting on doing it again	Self-identity	3.00 (86)	2.98 (66)	1.71 (0.42)	.032

^a^Miscellaneous topics comprised 8.00% (229/2867) of total tweets and 6.01% (133/2214) of spontaneous tweets and included positive evaluations of the intervention (3.00% (86/2867), eg, It's good to know there is a group of people going thru it w/me); reporting of stressful life events (eg, Have a cold. Chest hurts a lot); mentions of another’s support of the quit (eg, Oh and my hubby is still smoke free too! We're both on day 10); negative evaluations of the intervention (eg, I dunno how this Twitter stuff works); reporting of non-abstinence (eg, I'm still not all the way a non smoker. I've had a few this week); and other, each at about 1.01% (29/2867).

### Analyses

Models using generalized estimating equations (GEE; Proc Genmod, SAS v9.3) were run to assess the group effects (Group 1 versus 2) on abstinence status and nicotine patch use over time, after accounting for the clustering of participants within group, the time period (7, 30, or 60 days post quit date), and three participant-level covariates that the literature has identified as relating to abstinence success: education [25-28], gender [26,29], and baseline cigarettes per day [27,30,31]. We used similar models to assess the group effects on tweeting.

Additional models using GEE were run to assess how tweet volume, tweet content, and nicotine patch use related to abstinence status over time, after accounting for the effects of group, participants clustered within group, time period, and the three participant-level covariates. Separate models were estimated for total tweets, automessage-generated tweets, spontaneous tweets, each of the nine most common tweet types based on total tweets, and nicotine patch use. Abstinence at 7, 30, and 60 days post quit date was modeled as a function of the number of tweets of each type during the applicable time period (0-7 days, 8-30 days, and 31-60 days, respectively); thus, we used only the 2460 total tweets sent through day 60, which included 2023 spontaneous tweets (82.24%) and 437 automessage-generated tweets (17.76%).

## Results

### Participant Screening

Of the 813 smokers who completed the brief application form in response to our Google ads, 106 (13.0%) went on to complete the screening survey, and 45 of those (42.5%) met the eligibility criteria. The first 40 were chosen, and the other 5 were waitlisted. Ineligibility mostly occurred due to failure to complete the screening survey (21%), less than daily Facebook use (21%, Group 2), a phone that lacked Internet access (19%), lack of an unlimited texting plan (12%), smoking fewer than 5 cigarettes per day (12%), or texting less than weekly (10%). The eligibility rate was unrelated to gender or age but was higher for Caucasians (47%) than African Americans and Asian Americans (28%) and no Hispanics took the screener in this developmental trial.

### Participant Demographics and Smoking Histories

Participants averaged 36.5 years of age (SD 9.5, range 20-57), were 60% female, 95% Caucasian, 58% married or partnered, and 43% with a college degree. On average at baseline, participants smoked 15.5 cigarettes per day, had smoked for 18 years, and had a Fagerstrom nicotine dependency score of 4.9 indicating medium dependency [37]. The groups did not differ on any of these baseline variables (*P*=.187 to *P*=.667) except that Group 1 smoked marginally more cigarettes per day at baseline than Group 2 (mean 18.0 versus 13.0, *P*=.086), and thus cigarettes per day was included as a participant-level covariate in all models.

### Abstinence by Group

At 7, 30, and 60 days post quit date, Group 1’s abstinence rates were 50%, 57%, and 42% respectively; while Group 2’s abstinence rates were 21%, 61%, and 75% (*P*=.813). At the same time points, Group 1’s nicotine patch use rates were 67%, 50%, and 50% respectively, while Group 2’s use rates were significantly higher at 82%, 100%, and 42% (*P*=.019). In sum, the groups did not differ significantly on abstinence; however, Group 2 participants were more likely to use the study-provided nicotine patches.

### Tweeting by Group

**Figure 1:**
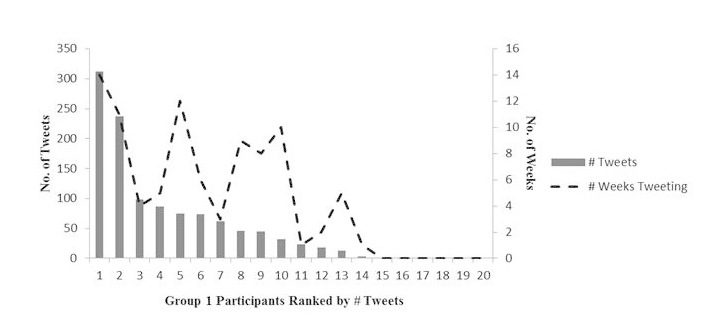
Tweeting volume and duration in Group 1.

**Figure 2:**
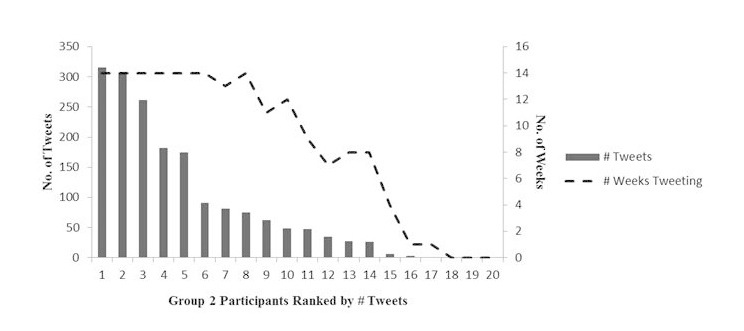
Tweeting volume and duration in Group 2.

Across the two groups, the total tweet volume was 2867 or an average of 72 tweets per group member; also 78% of the group members tweeted at least once. Automessages generated 22.78% (653) of the tweets, while the remaining 77.22% (2214) of the tweets were spontaneous. Figures 1 and 2 show tweeting volume and duration by group and participant.

Group 1 sent 1125 total tweets or an average of 56 tweets per member, while Group 2 sent 1742 total tweets or an average of 87 tweets per member (*P*=.355). Also 70% of Group 1 members and 85% of Group 2 members tweeted at last once (*P*=.121), and 45% of Group 1 members and 75% of Group 2 members continued tweeting past 30 days (*P*=.144). The groups differed significantly only in terms of their responses to the intervention automessages. Group 1 sent 51 automessage-generated responses or an average of 2.6 per member, while Group 2 sent 602 automessage-generated responses or an average of 30.1 per member (*P*<.001). This indicates that the developmental improvements made to the automessaging for Group 2 may have helped to increase responding to the automessaging.

**Figure 3:**
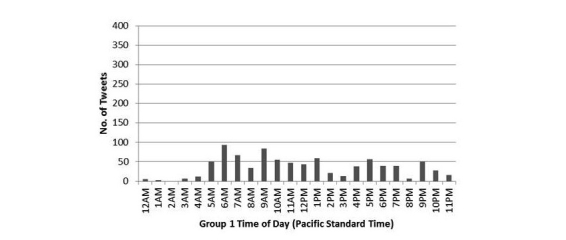
Tweeting by time of day in Group 1.

**Figure 4:**
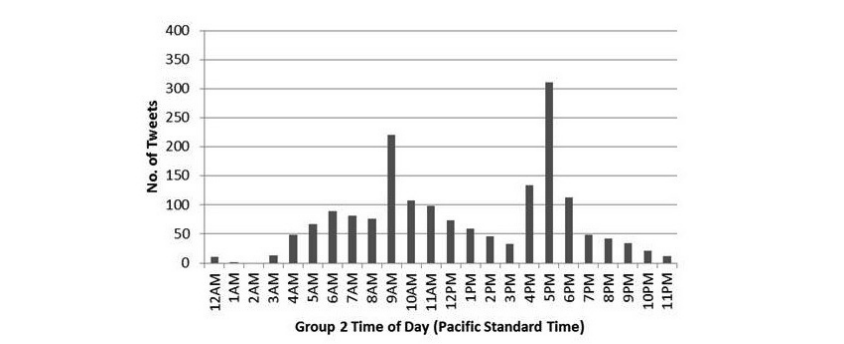
Tweeting by time of day in Group 2.

The groups were also compared on whether they showed spikes in tweeting that corresponded to the times when they were sent automessages and/or autofeedback (Figures 3 and 4). Group 1 members were sent automessages at 12 a.m. (midnight) Pacific, and autofeedback at 9 a.m. Pacific via Twitter, and they showed no time-related tweet spikes. In contrast, Group 2 members were sent automessages at 5 p.m. Pacific so that they could immediately respond, and they were sent autofeedback at 9 a.m. Pacific via text with no Twitter login required. Correspondingly, Group 2 showed tweet spikes after they were sent autofeedback and more markedly after they were sent automessages suggesting discussion topics.

### Content of Automessages and Automessage-Generated Responses

The automessages and automessage-generated responses were significantly correlated in terms of their content (*r*=.75, *P*=.012; Table 1). Looking at the automessage-generated responses, participants primarily shared smoking histories or other personal information (38%), identified rewards for quitting (18%), identified roadblocks to quitting (8%), or expressed confidence about quitting (6%). Also 11% of the automessage-generated responses discussed the intervention because so prompted.

Of the tweets that were coded as automessage-generated due to their timing, 6% provided questionable information about quitting (ie, non–evidence-based) and 5% asserted abstinence. This content was not prompted. Most of the questionable information about quitting mentioned using marijuana, e-cigarettes, candy, or food as a substitute for smoking rather than evidence-based substitutes such as exercise or relaxation.

### Content of Total Tweets

Total tweets and spontaneous tweets were highly correlated in terms of their content (*r*=.94, *P*<.001), that is, in terms of the number of tweets corresponding to each content code. Total tweets and automessage-generated tweets were less correlated (*r*=.57, *P*=.083), and spontaneous tweets and automessage-generated tweets were least correlated (*r*=.27, *P*=.444). Also, total tweets in Group 1 and Group 2 were highly correlated (*r*=.96, *P*<.001).

Prevalent content in the total tweets (Table 2) included sharing smoking histories or other personal information (24%), expressing emotional support for quitting (22%), asserting abstinence (12%), identifying roadblocks to quitting (10%), identifying rewards for quitting (8%), sharing questionable information about quitting (6%), or setting a quit date or using nicotine patches (4%). In effect, about 172 of the 2867 tweets contained questionable information about quitting (6.00%).

### Relationships Between Tweeting and Abstinence

In the models relating tweeting to abstinence, there were no group effects (*P*s>.420), but on the participant-level covariates, men were more likely to be abstinent (*P*s<.014). However, tests of treatment engagement by gender were all nonsignificant: men were not more likely than women to tweet, to tweet more, or to use the nicotine patch (*P*s>.500).

Abstinence related only marginally to overall tweet volume (OR 1.03, *P*=.086). Associations between abstinence and spontaneous tweet volume (OR 1.03, SE 0.02, *P*=.108) and automessage-generated tweet volume (OR 1.09, SE 0.08, *P*=.230) were not significant. Abstinence, however, was significantly related to sending tweets with this specific content (Table 2): assertions of abstinence (OR 1.17, SE 0.09, *P*=.031), setting of a quit date or use of nicotine patches (OR 1.52, SE 0.28, *P*=.024), countering of roadblocks to quitting (OR 1.76, SE 0.37, *P*=.008), and expressions of confidence about quitting (OR 1.71, SE 0.42, *P*=.032). Sending tweets about identifying rewards for quitting related only marginally to abstinence (OR 1.26, SE 0.16, *P*=.065).

Sending tweets with the following content did not relate to abstinence: sharing of smoking histories or other personal information (OR 1.08, SE 0.07, *P*=.237), expressions of emotional support for quitting (OR 1.04, SE 0.03, *P*=.156), identification of roadblocks to quitting (OR 1.02, SE 0.08, *P*=.754), or sharing of questionable information about quitting that was non–evidence-based (OR 1.12, SE 0.11, *P*=.278). Finally, nicotine patch use was unrelated to abstinence (OR 1.33, SE 0.66, *P*=.560).

## Discussion

### Principal Findings

In this developmental trial, we studied a novel, low-cost, fully automated social media-based smoking cessation intervention called Tweet2Quit. The intervention contained these components: smokers who were ready to quit were recruited online; they were placed in 100-day, 20-person, peer-to-peer Twitter support groups that were autonomous with no expert monitor; they were given free nicotine patches; and they were sent daily automessages suggesting discussion topics for tweeting and daily autofeedback on their prior day tweeting. This hybrid intervention combined the traditional social media approach of spontaneous, real-time, peer-to-peer social support with daily automessages that encouraged discussions consistent with guidelines for smoking cessation [12,13] and online community building [3,4].

Our first specific study aim was to explore if the automessages encouraged engagement and the findings look promising. Overall engagement in the intervention was high with 78% of the group members sending at least one tweet and each member sending an average of 72 tweets. Also 23% of the tweets were in response to the intervention’s automessages. Furthermore, when we improved the automessaging for Group 2 participants by increasing its frequency and improving its timing, automessage-generated responses rose significantly and we observed tweeting spikes corresponding to when the automessages were delivered. Moreover, the content of the automessages correlated with the content of the automessage-generated responses, indicating that the automessages largely produced the desired content. However, a randomized controlled trial is needed to study intervention efficacy.

Our second specific study aim was to assess whether overall engagement with the intervention or specific types of engagement were associated with abstinence. We found that the volume of overall tweets was only marginally related to the tweeter’s abstinence. However, the following specific types of tweets related significantly to the tweeter’s abstinence: countering of roadblocks to quitting, setting of a quit date or use of nicotine patches, expressions of confidence about quitting, and assertions of abstinence. Also identification of rewards for quitting was marginally related to abstinence.

Due to the correlational nature of our research, we cannot ascertain if specific types of tweets promoted abstinence; instead, perhaps abstinence elicited the tweeting. Nevertheless, it appears that automessages sent to online quit-smoking groups should try to encourage thinking and discussions on setting quit dates, using nicotine patches, countering roadblocks to quitting, building confidence about quitting, and identifying rewards for quitting. In our study, tweets that merely identified roadblocks to quitting were unrelated to abstinence, so it seems automessages should encourage participants to identify and counter roadblocks simultaneously.

In our study, expressions of emotional support for quitting (eg, “we can do this”) and the sharing of smoking histories or other personal information were unrelated to the tweeter’s abstinence; yet, these tweets may have promoted online community building [3,4]. Finally, we found that tweets conveying questionable or non-evidence-based information about quitting, for example, use of marijuana, e-cigarettes, or candy as substitutes for cigarettes, were in the minority (6%). Furthermore, these tweets were unrelated to the tweeter’s abstinence. Consistent with prior tobacco treatment studies [26,29], men were found to have higher rates of abstinence than women, and this appeared unrelated to treatment engagement. More research is needed to better understand gender differences in success with quitting smoking.

### Strengths and Limitations

This development trial was an important first step in exploring the utility of Twitter-based social support groups combined with automessages to promote smoking cessation. Limited by the nonrandomized treatment-only design, a randomized controlled trial is now needed. Also, our sample was small and primarily Caucasian. Although recruitment via Google was broad-based, offering nicotine patches as an incentive may have been more attractive to non-Hispanic Caucasian smokers given research that minorities are less likely to use patches [38,39]. Future research should test other approaches for engaging a more diverse group of smokers. Another study limitation was the reliance on self-reported abstinence, and so future research should test for bioconfirmation of abstinence, although the demand characteristics for false reporting of abstinence were likely low in our study due to the anonymity in the groups. Last, there were correlations among tweets addressing different content, making it difficult to fully assess the unique relationship between each content and abstinence.

### Conclusions

We have developed a hybrid social media–based smoking cessation intervention called Tweet2Quit that combines traditional real-time, peer-to-peer social support and nicotine patch with daily automessages and autofeedback. Engagement was high and the automessages helped ensure that the peer-to-peer discussions were consistent with guidelines for smoking cessation and building online communities. Hence, this novel intervention should be studied further.
